# Website Sharing in Online Health Communities: A Descriptive Analysis

**DOI:** 10.2196/jmir.5237

**Published:** 2016-01-13

**Authors:** Chinmoy Nath, Jina Huh, Abhishek Kalyan Adupa, Siddhartha R Jonnalagadda

**Affiliations:** ^1^ Division of Health and Biomedical Informatics Department of Preventive Medicine Northwestern University Feinberg School of Medicine Chicago, IL United States; ^2^ Department of Biomedical Informatics University of California San Diego San Diego, CA United States

**Keywords:** online systems, patient empowerment, online health communities, online health community moderators, consumer-health informatics, URLs, Web resources

## Abstract

**Background:**

An increasing number of people visit online health communities to seek health information. In these communities, people share experiences and information with others, often complemented with links to different websites. Understanding how people share websites can help us understand patients’ needs in online health communities and improve how peer patients share health information online.

**Objective:**

Our goal was to understand (1) what kinds of websites are shared, (2) information quality of the shared websites, (3) who shares websites, (4) community differences in website-sharing behavior, and (5) the contexts in which patients share websites. We aimed to find practical applications and implications of website-sharing practices in online health communities.

**Methods:**

We used regular expressions to extract URLs from 10 WebMD online health communities. We then categorized the URLs based on their top-level domains. We counted the number of trust codes (eg, accredited agencies’ formal evaluation and PubMed authors’ institutions) for each website to assess information quality. We used descriptive statistics to determine website-sharing activities. To understand the context of the URL being discussed, we conducted a simple random selection of 5 threads that contained at least one post with URLs from each community. Gathering all other posts in these threads resulted in 387 posts for open coding analysis with the goal of understanding motivations and situations in which website sharing occurred.

**Results:**

We extracted a total of 25,448 websites. The majority of the shared websites were .com (59.16%, 15,056/25,448) and WebMD internal (23.2%, 5905/25,448) websites; the least shared websites were social media websites (0.15%, 39/25,448). High-posting community members and moderators posted more websites with trust codes than low-posting community members did. The heart disease community had the highest percentage of websites containing trust codes compared to other communities. Members used websites to disseminate information, supportive evidence, resources for social support, and other ways to communicate.

**Conclusions:**

Online health communities can be used as important health care information resources for patients and caregivers. Our findings inform patients’ health information–sharing activities. This information assists health care providers, informaticians, and online health information entrepreneurs and developers in helping patients and caregivers make informed choices.

## Introduction

Increased access to online health information can empower patients to manage health better. A survey of US cancer patients showed that 92% of patients believed the Internet empowered them to make better health decisions and helped them communicate with their physicians [1]. Patients increasingly participate in online health communities and seek online health information; currently, more than 70,000 websites provide health information [2]. By May 2005, Yahoo! Groups [3] had listed more than 68,000 online support groups in their Health and Wellness section. Online health communities have been identified as one of the primary methods of online health information seeking for both consumers and members of their social networks [4-6]. Patients share their experiences and exchange emotional support and information through online health communities in the context of varying illnesses (eg, heart disease [7], rare diseases [8]). Patients also share resources for health information, including websites. Despite all the positive aspects of using online health information, it can be overwhelming, conflicting, and confusing for patients to find relevant, validated information [9]. Providing information to patients about the relevance and the validity of the websites posted in online health communities can assist in meeting the health information needs of patients while seeking online health information.

Members of online health communities, in addition to peer patients’ psychosocial support, increasingly share health information resources, such as links to websites. Gustafson et al [10] showed that informational support in online health communities has the potential to affect health care consumers’ decision making. Nambisan [11] studied the impact of empathy perceived by patients in an online health community based on their information-seeking effectiveness and social support. In online health communities, patients not only learn from peer patients, but also from online community moderators. Huh et al [12] showed that patients gained informational support from the community moderators. Further, other researchers have explored the assessment strategies for Internet information quality and readability [13], automated detection of conformity with the HONcode [14], computer-aided analysis of online social support [15], use of text mining and visualization for understanding smoking behavior [16], and analysis of top-level domain assignments [17].

These studies point to the importance of studying health information–sharing practices in online health communities. However, we lack knowledge around what kinds of information resources are being shared. One shared information resource in online health communities that we can easily capture is websites shared in the form of weblinks. We do not know what kinds of websites are being shared as an information resource and the context around how those resources are being shared.

Analyzing websites shared in online health communities should include the quality and purposes of these websites, who posts these websites, and whether there are any community differences. Investigating these issues around websites shared in online health communities will provide implications for developing how patients can appropriately navigate the online environment to locate relevant, high-quality health information.

Our research questions were:

1. Website categories: what kinds of websites are being shared in online health communities?
2. Information quality: what is the information quality of the websites being shared in online health communities?
3. Poster information: who are posting to those websites?
4. Community differences: how do communities post websites differently?
5. Context of website sharing: what are the contexts in which websites are being shared?

## Methods

### Data Collection

To answer our research questions, we chose the WebMD online health communities to investigate website-sharing practices. We chose WebMD because the community posts are publicly available and it is one of the most active online health communities online. We chose 10 WebMD online health communities on addiction, attention-deficit/hyperactivity disorder (ADHD), breast cancer, diabetes, weight loss, fit kids, heart disease, multiple sclerosis (MS), pain management, and sexual health. Our inclusion criteria for selecting these communities included being ranked within the top 15 communities in terms of total posting activity and having at least one health professional moderator and one staff moderator.

WebMD [18] is one of the few online health communities that offers both health professional and staff moderators. Health professional moderators at WebMD have clinical backgrounds in medicine, nursing, or nutrition. Staff moderators do not have clinical backgrounds, but facilitate and monitor conversations. We considered having enough moderator participation as criteria for choosing the community because we wanted to look at potential poster group differences in sharing websites.

We downloaded all posts from the 10 WebMD communities, which included 288,349 posts from June 2007 to February 2014. We received a letter from Northwestern University’s Institutional Review Board (IRB) that this study is not regulated by the IRB because our study is equivalent to the observation of public behavior.

### URL Extraction

To extract websites shared in WebMD online health communities, we extracted the URLs from each post using a regular expression pattern shown in the following:

“https?://[-w.]*(:d+)?

([w/_-.=?&%+@^~!#$]*)?

[^www]|www.(:d+)?

([w/_-.=?

&%+@^~!#$]*)?[^www].”

**Figure 1:**
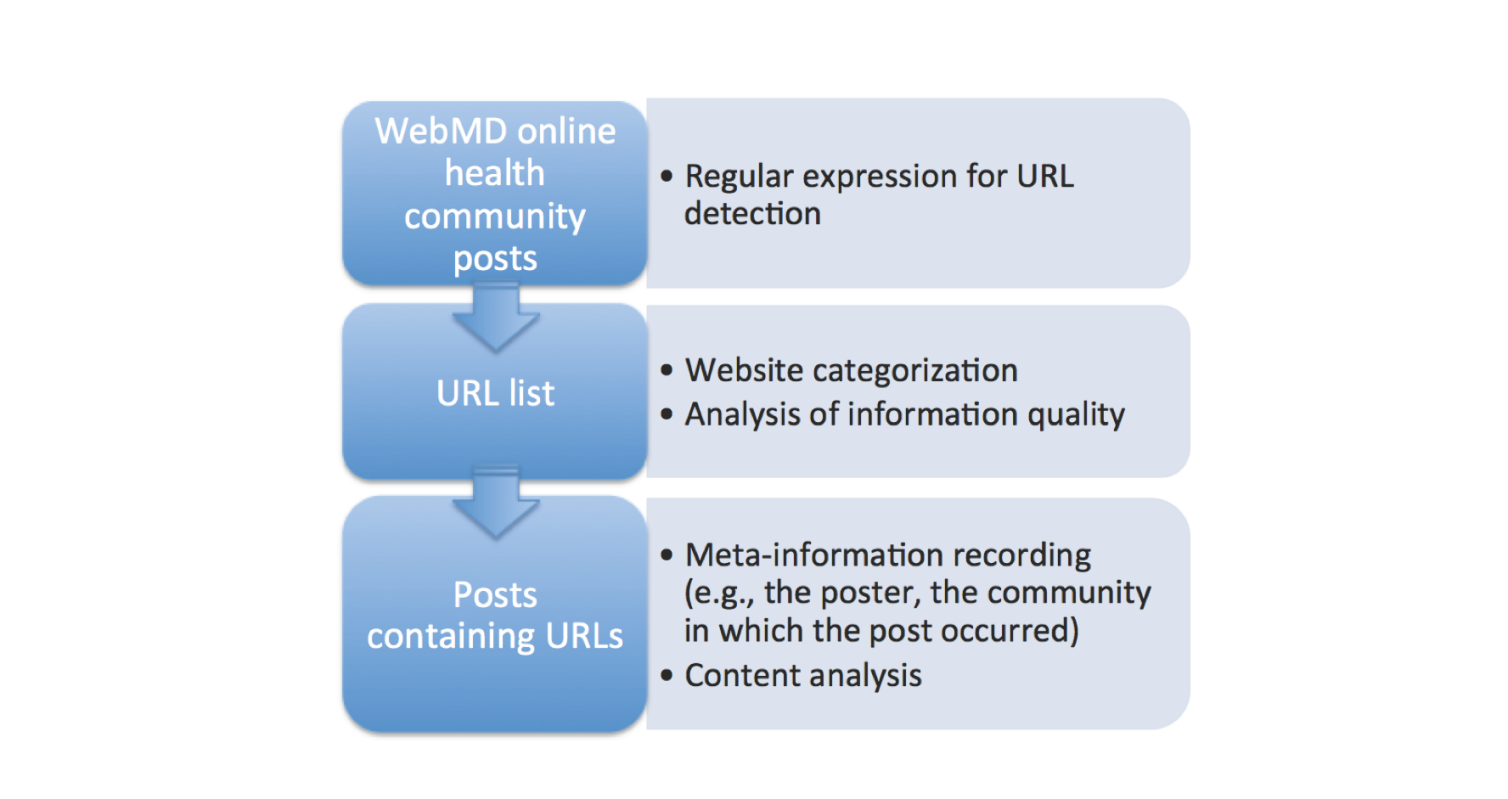
Process description from data collection to URL extraction and analysis.

Regular expressions are formal representations of text character patterns that represent a sequence of characters appearing in a text document with functionalities such as set operations (eg, union, intersection, negation), boundary matches, quantifiers (eg, at least once, exactly *n* times), and logical operators. We selected all posts containing at least one URL with this pattern. Many community members mentioned website names (eg, YouTube [19], Facebook [20]), but not the URLs linking to the website. We excluded such mentions of website names not following the conventional URL pattern as shown. Figure 1 shows the process of extracting and analyzing URLs in the dataset.

### Website Categorization

We then developed a mechanism to classify the websites identified from the URLs based on their top-level domain (TLD) names [21]. URLs and other resources connected to the Internet (servers, computer) are hierarchically separated by the dot (“.”) symbol. For example, the hierarchy for “en.wikipedia.org” is “org → Wikipedia → en.” TLDs are the top-most level in the hierarchy (org for “en.wikipedia.org”). Sometimes the TLDs are country code TLDs (ccTLD) (eg, “health.wa.gov.au”). In such cases, the ccTLD is ignored and the next domain name is considered as the TLD. We categorized a website as a “.gov” website if the TLD was .gov, an “.edu” website if the TLD was .edu, and a “.org” website if the TLD was .org. We also classified URL lists based on whether they could be considered as social media. It is difficult to identify social media websites from the domain names, let alone defining what a social media website is. For instance, Facebook is a representative website for social media. However, other generic websites, such as NYTimes.com, can also include social media features where the readers can interact online. To operationalize categorizing websites into social media, we selected the top 15 social media websites from eBizMBA [22], which included Facebook, Twitter, LinkedIn, Pinterest, Google Plus+, Tumblr, Instagram, VK, Flickr, Vine, Meetup, Tagged, Ask.fm, MeetMe, and ClassMates. For the URLs that contained these websites in their domain names (eg, www.facebook.com/pages/[...]), we categorized the URLs into “social media” websites.

### Analysis of Information Quality

To assess the quality of information shared in each website, we used the total number of trust codes assigned to the website. *Trust codes* refers to official validations the website has fulfilled in terms of health information quality requirements. The accredited agencies we used in our analysis conducting such validations included Health On the Net Foundation [23], True Ultimate Standards Everywhere Inc [24], Utilization Review Accreditation Commission [25], GuideStar USA Inc [26], National Committee for Quality Assurance [27], and National Health Council [28]. Accordingly, a website can have multiple validations through the form of trust codes that appears on their website, given by these agencies as evidence that they have fulfilled the requirements as a safe health information-sharing website.

Because of the overwhelming number of URLs extracted, we developed a systematic way to efficiently examine the information quality. If a website was mentioned 3 times or more from at least one WebMD community, we hand coded for assignment of trust codes. Then, we collected a list of commonly occurring keywords from the URLs of the websites identified to contain trust codes. Examples included “med,” “help,” “doc,” “Rx,” and “MD.” To assess the validity of the websites mentioned less than 3 times from one of the communities, we selected only those websites with URLs containing the previously listed keywords. Two authors (CN and AKA) hand coded trust codes for the websites that were mentioned at least 3 times or whose URLs contained these keywords. The interannotator agreement based on the kappa for assigning trust codes to these 1229 URLs was .948 (95% CI .932-.964), which is considered very good agreement [29]. We recorded the total number of trust codes for each website collected.

### Metainformation Recording: The Posters and the Community

To understand poster characteristics of website sharing, we aggregated community members into 3 groups: patient members, staff moderators, and health professional moderators. We used the list of staff moderators and health professional moderators’ usernames available on the WebMD website to identify these 3 poster groups. We then ranked all patient members based on their total posting frequency. We then subgrouped patient members as the following: (1) high-posting members (posters in the upper quartile of the list), (2) medium-posting members (posters in the interquartile of the list), and (3) low-posting members (posters in the lower quartile of the list). We also retained the information on which community the post came from (eg, diabetes vs heart disease).

### Qualitative Content Analysis

To qualitatively understand when and how community members shared URLs, we conducted a simple random selection of 5 threads among the conversation threads that included at least one URL in either the thread-initiating post or the replies from each of the 10 communities, resulting in a total of 50 threads. The number of replies to these threads varied between 2 and 15, resulting in a total of 386 posts for the qualitative analysis. We analyzed the post content using open coding analysis [30] for identifying emerging themes for sharing the URLs.

## Results

We extracted 25,448 URLs from 8714 unique posts out of the total 288,349 posts (3.02%) in all 10 communities (frequently shared websites shown in Multimedia Appendix 1). On average, posts in a community contained 1.99 (SD 1.14) URLs. Of all retrieved URLs, 94.83% (24,132/25,448) were posted in the replies.

Subsequently, we describe the categories of shared websites, the information quality of the shared websites, and findings around poster group and community differences in website-sharing behavior. We end with overall frequently shared websites and the context in which these URLs were shared.

### Results on Website Categories

**Figure 2:**
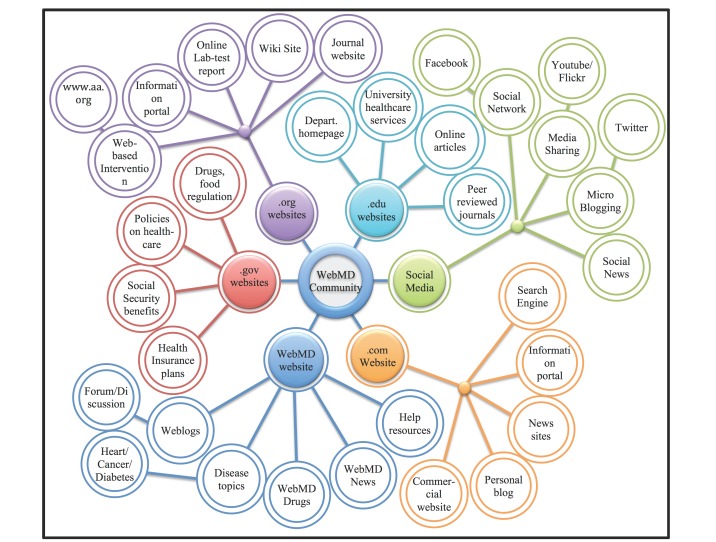
Trust code points across the website categories. The x-axis indicates the website categories and the y-axis indicates the percentage of websites with trust code points.

Our categorization criteria using TLDs resulted in 6 categories (in the order of appearance from high to low): .com websites, WebMD websites, .org websites, .gov websites, .edu websites, and social media websites. Figure 2 shows the website categories shared from our data and the content for each website category.

#### .com Websites

Out of the total of 25,448 URLs extracted, 15,056 URLs (59.16%) belonged to the .com websites. Websites classified in this category included search engines, information portal websites on drugs or medical tests, personal blogs, and commercial websites (eg, chemosavvy [31], Drugs.com [32], LIVESTRONG.COM [33], and Michelle’s Road to Recovery [34]).

#### WebMD Websites

In all, 5905 of 25,448 URLs (23.20%) belonged to the WebMD websites. Because our data came from the WebMD online health communities, those community members often shared resources they found from WebMD. To address this bias, we separated the URLs from the WebMD website as its own category, rather than including it as part of the .com websites. The identified WebMD websites included information on information on various diseases, drug information, news on health, or resources for crisis assistance.

#### .org Websites

Another 3369 of 25,448 URLs (13.24%) belonged to the .org websites. The URLs in this category included nonprofit organizations representing community members’ disease foci (eg, CHADD [35] for ADHD, Breastcancer.org [36] for breast cancer) and for-profit organizations related to the disease foci (eg, Joslin Diabetes Center [37] for diabetes). Other .org websites included wikis (eg, WikiEducator [38]), websites designed to help users understand laboratory test results (eg, Lab Tests Online [39]), journal websites (eg, American Medical Association [40], The American Journal of Clinical Nutrition [41]), and Web-based intervention websites.

#### .gov Websites

Of the 25,448 URLs, 930 (3.65%) belonged to government websites (eg, Centers for Disease Control and Prevention [CDC] [42], US Food and Drug Administration [FDA] [43]). These websites included information about government policies on health insurance plans, social security benefits, information on drugs, and food and health care.

#### .edu Websites

Another 149 of 25,448 URLs (0.59%) belonged to educational websites (eg, Perelman School of Medicine, University of Pennsylvania [44], University of South Florida [45]). Educational websites contained a university department’s website (introduction to the department), news related to innovative therapeutic research, online educational resources, and journal articles published by university faculty.

#### Social Media Websites

Finally, 39 of 25,448 URLs (0.15%) belonged to social media websites, including social networking sites such as Facebook, media-sharing apps such as YouTube [19] and Flickr [46], and microblogging sites such as Twitter [47].

### Information Quality of the Websites

#### Overall Information Quality

**Figure 3:**
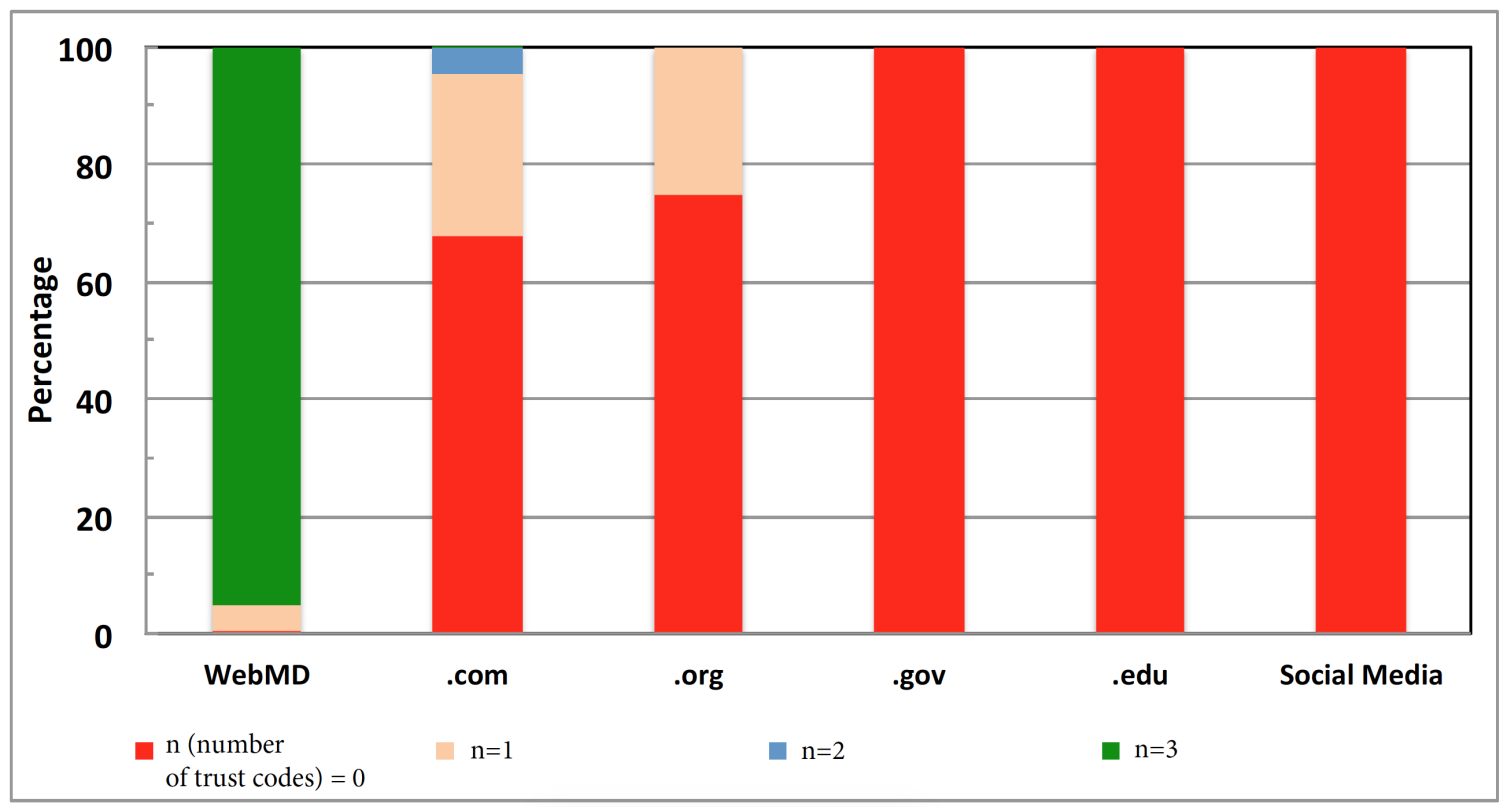
Trust code points across the website categories. The x-axis indicates the website categories and the y-axis indicates the percentages of websites with trust code points.

We found at least one trust code in 4875 URLs: 32.38% (1901/5872) of all URLs shared under the .com websites, 99.38% (5836/5872) of all URLs for the WebMD websites, and 25.20% (849/5872) of URLs for the .org websites (Figure 3). In terms of the average number of trust codes per URL, the .com websites had 0.36 trust codes per post (SD 0.57; n=15,056), 2.89 (SD 0.45; n=5872) for the WebMD website, and 0.25 (SD 0.43; n=3369) for the .org website.

For the rest of the website categories, all 930 URLs (100%) of the .gov websites, all 149 URLs (100%) of the .edu websites, and all 39 URLs (100%) of the social media websites did not contain any trust codes. A potential reason for this result is that social media websites contain information that can be posted without validation of their truthfulness.

To examine the quality of .edu websites with regards to their institutions’ expertise and existing work in disseminating health-related research, we investigated the number of systematic reviews published and indexed in PubMed. We focused on systematic reviews because they represent institutions with authors that synthesize evidence as opposed to focusing on primary literature. Our algorithm first retrieved the abstracts of all the 266,296 systematic reviews (as of November 11, 2015) using the clinical queries filter in PubMed [48]. Each abstract has an affiliation sentence that is often accompanied by the email address of the corresponding author(s). We used a simple regular expression (@?([^. <]+.)*[^. <]+) to extract the TLDs of the authors’ institution. We separated the .edu TLDs from this list and ranked the list as shown in Multimedia Appendix 2. We found that only 25 of 149 (16.8%) .edu URLs in our dataset were from educational institutions that did not have at least one systematic review.

We also separated the .gov TLDs from the list of TLDs extracted from systematic review affiliation sentences and ranked the list as shown in Multimedia Appendix 3. However, very few .gov institutions publish research and systematic reviews (eg, CDC, National Institutes of Health [NIH], Department of Veterans Affairs, Department of Health and Human Services, Agency for Healthcare Research and Quality). Many .gov websites (eg, cancer.gov and whitehouse.gov) that might contain reliable information are not in our list.

#### Poster Groups and Information Quality

There were 9671 high-posting members, 19,362 medium-posting members, and 9671 low-posting members. There were 88 staff moderators and 31 health professional moderators.

**Figure 4:**
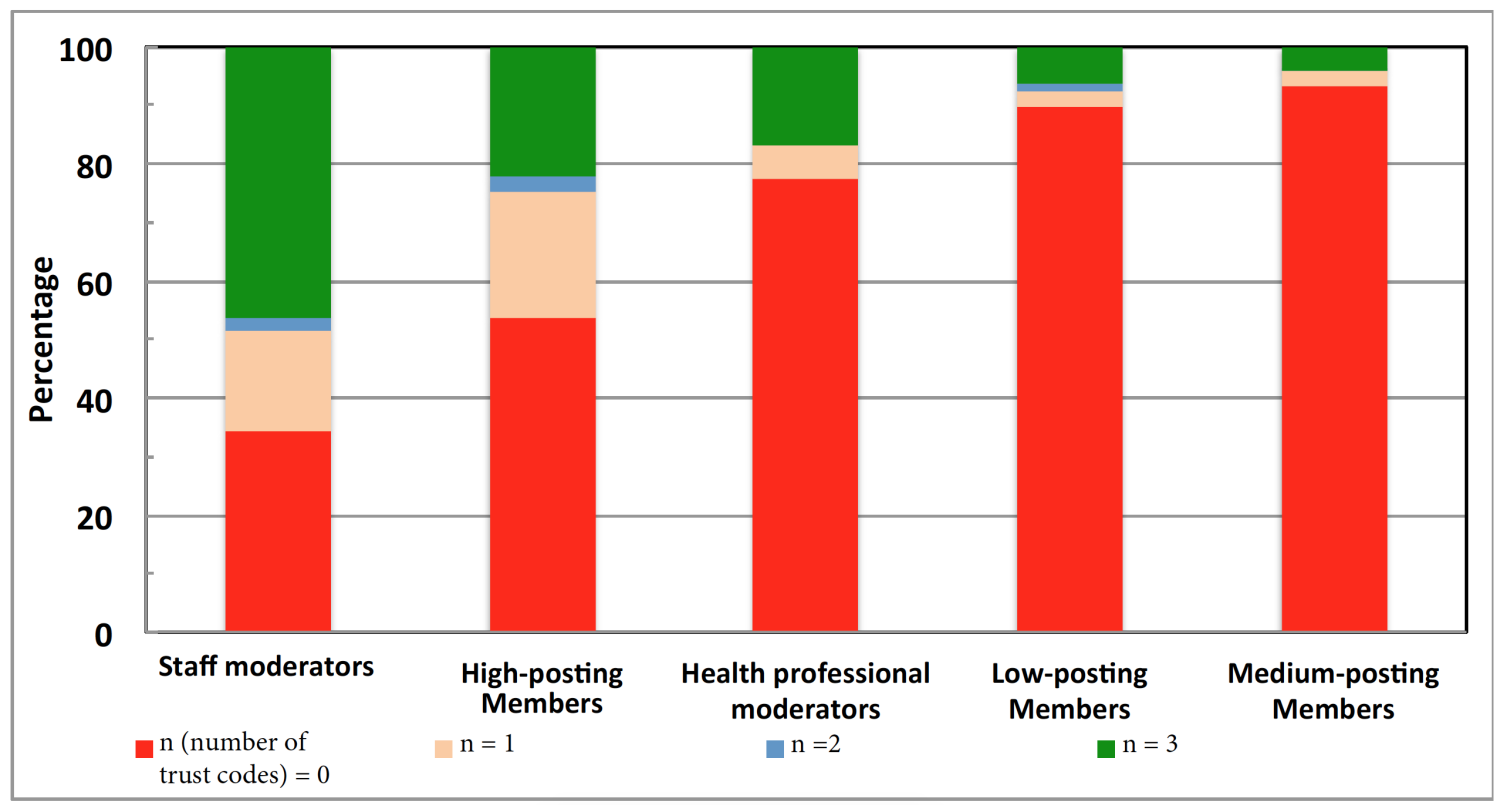
Trust code points among various member groups. The x-axis indicates the member groups and the y-axis indicates the percentage of website with trust code points.

We found at least one trust code in 46.11% (4459/9671) of all URLs posted by the high-posting members, 6.67% (1911/19,362) for the medium-posting members, and 10.11% (978/9671) for the low-posting members. For the moderators, we found 66% (58/88) of all URLs posted by staff moderators and 23% (7/31) for the health professional moderators contained at least one trust code (Figure 4). The average trust code numbers per URLs shared followed the same order: the staff moderators ranked the highest (n=474 trust codes; mean 1.61, SD 1.36), followed by high-posting members (n=24,252; mean 0.92, SD 1.20), health professional moderators (n=180; mean 0.56, SD 1.12), low-posting members (n=188; mean 0.24, SD 0.77), and medium-posting members (n=360; mean 0.15, SD 0.60).

#### Community Differences and Information Quality

Table 1 shows the total number of posts, the number of posts containing URLs, and the mean number of URLs per post for each community. In the heart disease community, 3107 of the total 14,033 posts (22.14%) contained at least one URL. In other WebMD communities, less than 6% (mean 2.71%, SD 1.47%) of total posts contained URLs. On average, the heart disease community shared more than one URL per post, whereas other WebMD communities shared fewer than one URL per post. The heart disease community’s total number of posts were fewer than many of the other communities. However, the total number of URLs shared in the heart disease community alone (n=16,146) was more than all URLs shared combined in other communities (n=9315).

**Table 1:** Total number of posts, posts containing URLs, URLs, and mean URLs per post for each WebMD community.

Community	Total posts	Posts containing URLs, n (%)	Total URLs	URLs per post
Heart	14,033	3107 (22.14)	16,146	1.15
Diabetes	71,168	2079 (2.92)	3586	0.05
Weight loss	58,344	956 (1.64)	1474	0.03
Breast cancer	26,653	729 (2.74)	1376	0.05
Sexual health	68,113	677 (0.99)	849	0.01
MS	28,267	527 (1.86)	848	0.03
ADHD	9637	363 (3.77)	697	0.07
Pain	8108	203 (2.50)	373	0.05
Addiction	3806	61 (1.60)	95	0.02
Fit kids	220	12 (5.5)	17	0.08

**Figure 5:**
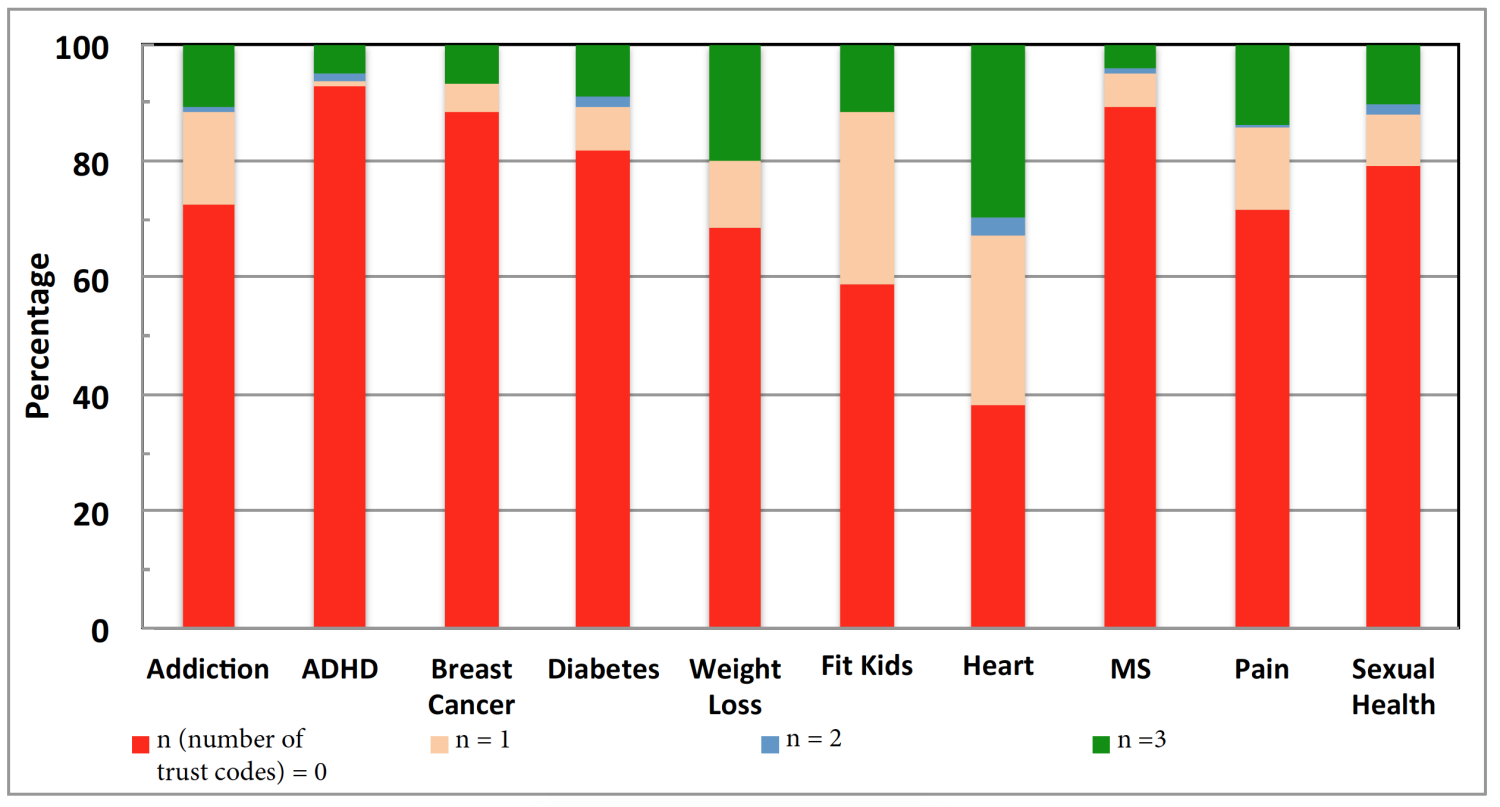
Trust code points in various online health communities. The x-axis indicates the online health communities and the y-axis indicates the percentage of websites with trust codes points.

For information quality, the heart disease community had the highest percentage of URLs containing at least one trust code (61.60%, 9947/16146). The next in line was the fit kids community (41%, 7/17), followed by the weight loss community (31.47%, 464/1474), the pain community (28.4%, 106/373), the addiction community (27%, 26/95), the sexual health community (20.8%, 177/849), the diabetes community (18.07%, 648/3586), the breast cancer community (11.7%, 66/566), the MS community (10.5%, 89/848), and the ADHD community (7.2%, 50/697) (Figure 5). The mean trust codes per post was highest for the heart community (n=16,146 trust codes; mean 1.23, SD 1.24). The next in line was the weight lost community (n=1474; mean 0.71, SD 1.18), followed by the fit kids community (n=17; mean 0.65, SD 0.97), the pain community (n=373; mean 0.56, SD 1.04), the addiction community (n=95; mean 0.49, SD 0.95), the sexual health community (n=849; mean 0.43, SD 0.95), the diabetes community (n=3586; mean 0.38, SD 0.90), the breast cancer community (n=566; mean 0.25, SD 0.78), the MS community (n=848; mean 0.19, SD 0.65), and the ADHD community (n=697; mean 0.18, SD 0.69).

### Content Analysis

Within our dataset for content analysis, some threads had more than one post containing URLs. There were 64 posts that contained URLs (mean 1.28, SD 2.06 posts containing URLs per thread). Members used URLs in a number of ways: to disseminate information (41/64, 64%), to find alternative means of communication (5/64, 8%), to extend social support (6/64, 9%), and to use as a resource for supporting evidence (12/64, 18%). Of the 64 posts containing URLs, 13 were posted by the WebMD moderators, who mostly did so to disseminate information (7/13, 54%) and help members extend social support (4/13, 31%). WebMD moderators also posted URLs as a resource for supporting evidence (2/13, 15%). Only 3 of 64 (7%) URLs containing posts were thread-initiating posts, showing that members in our content analysis sample shared URLs to respond to others’ questions, rather than voluntarily disseminating information.

“Disseminate information” was the most frequently occurring theme. This theme included solutions and answers to questions, news, new study findings, product information, and education materials for new members. For instance, a member in the WebMD pain management community initiated a thread regarding an article against the petition to the FDA to modify the label on opioid medications. The petition called for constraining opioid use for non-cancer patients, which would be of concern to those dealing with pain. The members developed the thread into a heated debate about the effectiveness of such petitions.

Another example of disseminating information occurred when members asked questions that showed their early status in their illness and other members shared resources that new patients needed to know. In the WebMD breast cancer community, a member posted that she was just told to either have a biopsy or have a lump removed. To this post, another member responded with URLs regarding information about insurance as well as websites (eg, breastcancer.org) and phone numbers the member could call to get additional help on being newly diagnosed with breast cancer.

The members also posted URLs that allowed alternative means of communication, such as a separate forum or thread to share experiences. In the WebMD fit kids community, a member posted a WebMD forum link through which other members could share their ideas on, for instance, cooking with kids to help them make healthy eating choices. A member started a thread with the following message: “The topic is cooking with kids! How do you have fun with your kids in the kitchen and help them learn about healthy choices? Share your favorite ideas and recipes here! [URL]”

To those posters who were seeking social support, members responded with URLs through which the members could seek further emotional and informational support. For instance, in the WebMD sexual health community, a member posted her concerns that her fiancé was positive for the human immunodeficiency virus (HIV) and, although she loves him, she is concerned about their future sexual health. To this post, another member provided a link to an HIV community, stating that she could receive better support in that community.

Lastly, members used URLs to support, add, or clarify their arguments stated in their posts. Standards, guidelines, and definitions (eg, food portions for kids) were shared from authoritative, government websites. Members also used research articles listed on PubMed as evidence when they talked about the efficacy of certain treatments. Among the repliers, sometimes URLs were used to debate opposite opinions. For instance, in the ADHD community on WebMD, members debated how medications were harmful or benign based on research study findings linked with URLs.

In summary, members used URLs in many ways that triggered conversations, enriched their discussions, supported arguments, and added validity to various types of information shared.

### Frequently Shared Websites

Of the top 50 frequently shared websites across all communities, 86% (43/50) contained health information (eg, www.hrspatients.org, www.healingwell.com); 50% (21/43) of these health information-providing websites were either .gov websites or websites certified with trust codes (eg, NIH [49]). Of the top 50 ranked websites, 54% (27/50) belonged to the .com website category, 4% (2/50) were blog websites (eg, The Life of Teddybear’s Owner [50]), 2% (1/50) were social media websites (eg, YouTube [19]), and another 2% (1/50) were .org websites (eg, Northwestern Medicine [51]). All these websites are included in Multimedia Appendix 1.

Table 2 shows the top 10 frequently shared websites. These websites included 6 .com websites, 3 .org websites containing at least one trust code, and one .gov website. The top 10 websites belonging to the .com websites included information on health and health care, drugs and pharmacy information, community support, and repositories of personal medical information and healthy lifestyle information, including food, nutrition, and physical exercise. The .org websites belonged to a health care institution and disease-specific nonprofit organizations. The .gov website included in this list was a leading public health institute conducting research and providing information for control and prevention of diseases.

**Table 2:** The top 10 most-shared websites.

Websites	Occurrences, n
WebMD [18]	1739
Mayo Clinic [52]	544
HeartSite.com [53]	446
HealingWell.com [54]	290
myOptumHealth [55]	216
Heart Rhythm Society [56]	112
American Heart Association [57]	104
FITDAY [58]	94
ehealthMD [59]	89
US National Library of Medicine [60]	88

## Discussion

In this paper, we present the website-sharing practices of online health community users. Using objective measures, such as post frequency, TLD, and trust code assignment, we learned about the kinds of websites shared, the information quality of these websites, the posters of these websites, and the community differences. We also show the context in which these websites were shared. Subsequently, we discuss the implications and practical applications of our findings.

### Website Categories: .com Websites and Internal Resource Use

The majority of websites shared were .com websites. The community members rarely shared links to the popular social media websites. The .com websites contained a variety of content areas spanning from news, access to portal, and to personal blogs. Approximately one-third of these .com websites had at least one trust code assignment, meaning that at least one-third of these .com websites belonged to validated health information–sharing websites. Considering that the majority of websites shared were .com websites, more sophisticated methods to detect the content of the websites will help us understand the kinds of information community members attempt to share. For instance, the TLD can be further analyzed to understand whether it contains health-related keywords. The content on the main page can be scraped and automatically analyzed to generate topic distributions of the websites shared in online health communities. This information can then inform community members as well as researchers and practitioners whose goal is to develop better systems that can help patients gain high-quality information.

WebMD websites were ranked as the second most-shared website. This finding shows that the community members increasingly used the resources housed in their parent website. This finding shows the importance in choosing the parent website environment for establishing online health communities. The information quality of the websites shared in online health communities can be influenced by the quality of the parent website.

### Information Quality: Rethinking Information Quality Detection

For this study, we focused on objective, efficient methods to understand website-sharing practices. The scope of our technique involved using (1) the posting frequency to understand the overall prevalence of various website-sharing practices, (2) the TLDs, which is extremely limited information, to categorize websites, and (3) the assignment of trust codes to assess the information quality. Our approach was helpful in gaining objective and efficient assessment over information quality. However, we faced a few difficulties in detecting the quality of .gov, social media, and .edu website categories. In the case of WebMD websites and other health information portals, their primary goal was to deliver health information to patients. Such health websites inevitably need to add trust codes to reassure that the visitors understand the quality of the website. The .gov and .edu websites are not found to have accreditations in general, but they might be trusted when associated with institutions with solid reputations [61]. None of the social media websites contained trust codes because they do not have an obligation to validate their health information quality; their primary focus is not necessarily sharing health information.

To further develop automated information quality detection, we need to rethink what is high-quality information.

Our measurement of information quality of the shared websites does not address potential unanticipated benefits that websites without trust codes can provide patients. For instance, Nambisan [11] showed that the key gratification for patients from online health communities is perceived empathy. Perceived empathy has the potential to directly affect the success of the treatment and it could supplement a caregiver’s provided empathy, which is expensive and time consuming. Choi [62] reported that people increasingly share information about health care institutions through videos. She reported that traffic from YouTube to hospital sites increased 119% over a year in the 2012 Google/Compete Hospital Study; 30% of patients who watched a video made an appointment with that hospital.

Future research should investigate information quality methods for each website category and contexts in which websites are shared. Our qualitative analysis of the website-sharing context indicated how information quality only matters half of the time people share websites—only when they want to disseminate information and use websites as supportive evidence. We need systems that would identify these varying needs before making uniform decisions about information quality of the shared resources.

In some threads, exchanges of appreciation and greetings took place after members shared websites. In such situations, the website itself acted as a catalyst for social networking among members. Members also shared websites linked with social media as a platform for sharing health information. Members posted Facebook webpages restricted for chemotherapy patients sharing various experiences and information, social medial profiles maintained by independent organizations to assist decision making in medical care, and websites maintained by research groups to assist others in exploring advanced health care topics. Social media websites can play an important role in disseminating what we traditionally consider “validated information” along with empowering anecdotes.

### Poster Differences: Activity and Role of the Poster and Information Quality

Depending on how active the poster is, the quality and quantity of websites can differ. High frequency posters and moderators shared higher number of websites assigned with trust codes compared to the medium- to low-posting posters. It could be that the high-posting users and moderators share more health information-related websites than the lower posting users. Another explanation is that high-posting users and moderators take on the information dissemination role, which forces them into sharing validated health information websites. Fox et al [63,64] showed that the more experienced an Internet user is, the more likely they will search for health information online. Oh [65] showed that altruism is the most influential motivation and personal gain is the least motivating factor for responders to health questions in online health communities. Accordingly, because of the altruistic motivation, high-posting community members might be motivated to make sure they share Web resources with high-quality information. When developing information quality assessment tools or guidelines for online health communities, our findings inform it is important to take into account the posting frequency of posting members.

### Community Differences: Disease Differences and Information Needs

We learned that the heart community shared the highest number of websites and the most websites with trust codes. Heart disease, with its potential to generate urgency in treatment, might push the community members to share websites that focus on validated health information. On the other hand, the ADHD community shared the least proportion websites with trust codes. Patients and caregivers with ADHD often face disagreement with their providers regarding diagnosis and treatment [66]. Thus, patients and caregivers of ADHD patients might share more controversial information sources. The addiction community shared several websites providing online intervention programs. In the weight loss community, diet and nutrition intervention programs were shared. Similarly, in the sexual health community, websites with intervention for sex-addicted patients were shared. Christakis and Fowler [67] showed that smoking and alcohol cessation programs and weight loss interventions that provide peer support (ie, that modify the person’s social network) are more successful than those that do not. Depending on the disease and the patients’ relationship and existing challenges around health care could be reflected in their website-sharing practices. Again, the definition of what is high information quality in online health communities is highly situated.

### Addressing Situated Quality: Practical Applications for the Stakeholders

Our findings inform a number of stakeholders, including health care practitioners, patients and caregivers, researchers, and online health information system entrepreneurs and developers. We discuss how situated quality should be addressed in health information sharing in online health communities.

The health care practitioners can learn from our frequently shared websites and descriptive results about what kinds of information patients navigate through. Based on our findings, health care practitioners can either redirect or encourage their patients about the websites they should be cautious of or further investigate. Patients and caregivers can use our findings to guide their future use of online health communities and think about what provisions should be made when using online health communities.

Researchers should further examine ways to improve information quality detection and understanding situated quality, the information quality that is a suitable guideline depending on the disease context and the motivation for sharing information. The online health community entrepreneurs and developers should think about the following when helping to improve information sharing practices in online health communities:

1. Develop real-time assessment of the categories and information quality of shared websites using our techniques: this information can be used for moderators in improving quality of posts.
2. Develop ways to further categorize .com websites in a meaningful manner.
3. Develop a situated information quality assessment tool based on poster characteristics, TLDs, trust codes, and context of posting (eg, thread initiator post vs reply).
4. Aggregate and summarize all websites for all community members to use.
5. In sharing summarized list of websites, reflect the situated context of the posts in which the websites came from.

One of the limitations of this study is that we were unable to collect demographic information on the patients because of WebMD privacy settings. Such patient profiles can further add the situated needs in why patients share websites. Also, many members posted the website’s name alone without mentioning URLs. Our algorithm ignored websites that did not follow the regular expression pattern we designed. Because of our semiautomated search for trust codes on websites, it is possible we missed that some of the websites included trust codes. More sophisticated information quality assessment methods can be developed using our findings.

### Conclusions

Online health communities have emerged as one of the core places that patients visit to gain health care information resources and social support. We observed that sharing websites played a vital role in building networks among members of online health communities. We analyzed different contexts under which website sharing takes place and how different Web resources serve members’ informational and emotional needs. We summarized the most frequent Web resources disseminated over 10 online health communities. Health care practitioners, content developers, and informaticians can use our findings to further understand how patients share websites online. Our findings might help these stakeholders to design systems that can help patients and caregivers make more informed choices.

## Appendix

Multimedia Appendix 1 List of top 50 websites shared in ten different online health communities.

Multimedia Appendix 2 Educational websites sorted by frequency of systematic review email addresses.

Multimedia Appendix 3 Government websites sorted by frequency of systematic review email addresses.

## Abbreviations

ADHD: attention-deficit/hyperactivity disorder
ccTLD: country code top-level domain
CDC: Centers for Disease Control and Prevention
FDA: US Food and Drug Administration
HIV: human immunodeficiency virus
IRB: Institutional Review Board
MS: multiple sclerosis
NIH: National Institutes of Health
TLD: top-level domain

## References

[1] McMullan M (2006). Patients using the Internet to obtain health information: how this affects the patient-health professional relationship. Patient Educ Couns.

[2] Grandinetti D (2000). Doctors and the Web. Help your patients surf the Net safely. Med Econ.

[3] Yahoo! Groups.

[4] Kummervold PE, Gammon D, Bergvik S, Johnsen JK, Hasvold T, Rosenvinge JH (2002). Social support in a wired world: use of online mental health forums in Norway. Nord J Psychiatry.

[5] Cline RJ, Haynes KM (2001). Consumer health information seeking on the Internet: the state of the art. Health Educ Res.

[6] Cotten S (2001). Implications of Internet technology for medical sociology in the new millennium. Sociol Spectrum.

[7] Bonniface L, Green L (2007). Finding a new kind of knowledge on the HeartNET website. Health Info Libr J.

[8] Coulson NS, Buchanan H, Aubeeluck A (2007). Social support in cyberspace: a content analysis of communication within a Huntington's disease online support group. Patient Educ Couns.

[9] Eysenbach G (2003). The impact of the Internet on cancer outcomes. CA Cancer J Clin.

[10] Gustafson DH, Hawkins R, Pingree S, McTavish F, Arora NK, Mendenhall J, Cella DF, Serlin RC, Apantaku FM, Stewart J, Salner A (2001). Effect of computer support on younger women with breast cancer. J Gen Intern Med.

[11] Nambisan P (2011). Information seeking and social support in online health communities: impact on patients' perceived empathy. J Am Med Inform Assoc.

[12] Huh J, McDonald D, Hartzler A, Pratt W (2013). Patient moderator interaction in online health communities. AMIA Annu Symp Proc.

[13] Hanna K, Brennan D, Sambrook P, Armfield J (2015). Third molars on the Internet: a guide for assessing information quality and readability. Interact J Med Res.

[14] Boyer C, Dolamic L (2015). Automated detection of HONcode website conformity compared to manual detection: an evaluation. J Med Internet Res.

[15] Wang Y, Kraut R, Levine J (2015). Eliciting and receiving online support: using computer-aided content analysis to examine the dynamics of online social support. J Med Internet Res.

[16] Chen AT, Zhu S, Conway M (2015). What online communities can tell us about electronic cigarettes and hookah use: a study using text mining and visualization techniques. J Med Internet Res.

[17] Eysenbach G (2014). The new health-related top-level domains are coming: will cureforcancer.health go to the highest bidder?. J Med Internet Res.

[18] WebMD.

[19] YouTube.

[20] Facebook.

[21] Postel J (1994). RFC 1591: Domain Name System Structure and Delegation.

[22] (2015). eBizMBA.

[23] Health on the Net Foundation.

[24] True Ultimate Standards Everywhere, Inc.

[25] Utilization Review Accreditation Commission.

[26] GuideStar.

[27] National Committee for Quality Assurance.

[28] National Health Council Standards of Excellence Certification Program.

[29] Fleiss J, Levin B, Paik M (2003). Statistical Methods for Rates and Proportions.

[30] Corbin J, Strauss A (1990). Grounded theory research: procedures, canons, and evaluative criteria. Qual Sociol.

[31] ChemoSavvy.

[32] Drugs.com.

[33] LIVESTRONG.COM.

[34] Michelle’s Road to Recovery.

[35] CHADD: The National Resource on ADHD.

[36] Breastcancer.org.

[37] Joslin Diabetes Center.

[38] WikiEducator.

[39] Lab Tests Online.

[40] American Medical Association.

[41] The American Journal of Clinical Nutrition.

[42] Centers for Disease Control and Prevention.

[43] US Food and Drug Administration.

[44] Perelman School of Medicine University of Pennsylvania, Department of Psychiatry, Penn Behavioral Health.

[45] TRIGR (Trial to Reduce IDDM in the Genetically at Risk).

[46] Flickr.

[47] Twitter.

[48] Haynes RB, Wilczynski N (2005). Finding the gold in MEDLINE: clinical queries. ACP J Club.

[49] National Institutes of Health.

[50] The Life of Teddybear’s Owner.

[51] Cadence Health.

[52] Mayo Clinic.

[53] Meystre S, Haug PJ (2006). Natural language processing to extract medical problems from electronic clinical documents: performance evaluation. J Biomed Inform.

[54] HealingWell.com.

[55] myOptumHealth.

[56] Heart Rhythm Society.

[57] American Heart Association.

[58] FitDay Free Diet and Weight Loss Journal.

[59] Demner-Fushman D, Chapman WW, McDonald CJ (2009). What can natural language processing do for clinical decision support?. J Biomed Inform.

[60] US National Library of Medicine.

[61] (2014). National Institute on Aging.

[62] Choi M (2015). Social media in clinical practice. Healthcare Inform Res.

[63] Fox S, Fallows D (2003). Pew Research Center.

[64] Fox S, Rainie L (2000). Pew Research Center.

[65] Oh S (2011). The characteristics and motivations of health answerers for sharing information, knowledge, and experiences in online environments. J Am Soc Inf Sci.

[66] Leslie LK, Plemmons D, Monn AR, Palinkas LA (2007). Investigating ADHD treatment trajectories: listening to families' stories about medication use. J Dev Behav Pediatr.

[67] Christakis NA, Fowler JH (2009). Social network visualization in epidemiology. Nor Epidemiol.

